# Effects of oral Lcarnitine supplementation on liver enzymes in pediatric acute lymphoblastic leukemia patients in the maintenance phase of treatment: a randomized clinical trial study

**DOI:** 10.3389/fphar.2024.1507996

**Published:** 2025-01-17

**Authors:** Aziz Eghbali, Aygin Eghbali, Neda Ashayeri, Fatemeh Fadayi, Kazem Ghaffari, Ali Ghasemi

**Affiliations:** ^1^ Clinical Research Development Center of Aliasghar Hospital, Iran University of Medical Sciences, Tehran, Iran; ^2^ School of Medicine, Iran University of Medical Sciences, Tehran, Iran; ^3^ Department of Hematology and Blood Transfusion Sciences, School of Allied Medical Sciences, Tehran University of Medical Sciences, Tehran, Iran; ^4^ Department of Basic and Laboratory Sciences, Khomein University of Medical Sciences, Khomein, Iran; ^5^ Student’s Scientific Research Center, Tehran University of Medical Sciences, Tehran, Iran; ^6^ Cancer Research Center, Semnan University of Medical Sciences, Semnan, Iran; ^7^ Department of Biochemistry and Hematology, Semnan University of Medical Sciences, Semnan, Iran

**Keywords:** Lcarnitine, liver enzymes, pediatric, acute lymphoblastic leukemia, chemotherapy

## Abstract

**Background:**

Given that liver diseases and subsequent increases in liver enzymes are among the most frequent complications observed in leukemia patients treated with chemotherapeutic drugs, this study aimed to assess the impact of oral Lcarnitine supplementation on liver enzyme levels the maintenance phase of treatment for pediatric acute lymphoblastic leukemia (ALL) patients.

**Methods:**

In this clinical trial, 100 pediatric patients aged >5 years were divided into two groups after obtaining informed consent. The participants were randomly divided into the Lcarnitine and placebo groups. In the Lcarnitine group, patients received 50 mg/kg of Lcarnitine syrup three times a day (every 8 h). Patients were examined for 2 months to receive Lcarnitine syrup and to measure the levels of alanine aminotransferase (ALT), aspartate transferase (AST), alkaline phosphatase (ALP), gamma-glutamyl transferase (GGT), direct bilirubin, total bilirubin, prothrombin time (PT), and partial thromboplastin time (PTT).

**Results:**

The mean changes in AST, ALT, total bilirubin, and GGT during the study period were significant in the group treated with Lcarnitine (P < 0.05), although they were not significant in the placebo group (P > 0.05). Also, the levels of ALP, direct bilirubin, PT, and PTT were not significantly different between the two groups. The incidence of side effects was significantly higher in the Lcarnitine group than in the placebo group (18% vs 4%, P = 0.025).

**Conclusion:**

The results of this study suggested that a 60-day Lcarnitine treatment can improve liver enzyme levels and thus prevent the extent of liver damage during the treatment of ALL. Based on the results of our study, Lcarnitine supplementation may have a beneficial effect on liver enzyme levels in pediatric ALL patients during the maintenance phase of treatment.

**Clinical Trial Registration:**

https://irct.behdasht.gov.ir/search/result?query=IRCT20201107049296N2, identifier IRCT20201107049296N2

## 1 Introduction

Cancer is a significant contributor to mortality rates in both developed and developing countries and often manifests abruptly. Pediatric and adolescent cancer typically manifests from birth until the age of 19 years and is classified as a life-threatening condition (Seiler and Jenewein, 2019; Hadadhania et al., 2023; Ghasemi et al., 2022; Mortazavi et al., 2024). Leukemia is a cancer affecting blood cell production in the bone marrow, characterized by an abnormal increase in leukocytes (Ghasemi et al., 2016; Ghaffari et al., 2023a; Ghaffari et al., 2023b; Ghasemi et al., 2015; Ghasemi et al., 2020).

Approximately 25% of pediatric cancer diagnoses are associated with acute lymphoblastic leukemia (ALL), indicating that ALL is the most prevalent form of cancer in the pediatric population (Eghbali et al., 2023a; Ghaffari et al., 2022a). The highest incidence of ALL is observed in children aged 2–5 years, with an annual prevalence rate of 36.2 per 1 million individuals (Inaba and Pui, 2021; Ghaffari et al., 2022b). In Iran, hematological malignancies are among the sixth most prevalent types of cancer affecting both sexes (Dastgiri et al., 2011).

Chemotherapy, whether administered as monotherapy or in conjunction with alternative therapeutic modalities, continues to be the primary treatment for ALL (Sheykhhasan et al., 2022; Eghbali et al., 2023b). In the context of pediatric ALL patients, the administration of chemotherapeutic agents is frequently correlated with the occurrence of hepatotoxicity (Ladas et al., 2010; Eghbali et al., 2023c). Consequently, the delivery of such chemotherapeutic agents is often suspended, particularly during the maintenance phase of the treatment regimen. A recent investigation indicated that a substantial proportion of patients with ALL—exceeding fifty percent—encounter grade 2 or greater hepatotoxicity during the maintenance treatment phase (Farrow et al., 1997; Eghbali et al., 2024). Conversely, cessation of chemotherapeutic agents increases the risk of relapse in bone marrow (Ladas et al., 2010; Matutes et al., 1991; Fehér and Lengyel, 2012; Schmiegelow, 1991). Among the array of chemotherapeutics, methotrexate and 6-mercaptopurine are used during the maintenance phase of ALL treatment, both of which are implicated in hepatotoxicity. Furthermore, varying degrees of hepatic fibrosis have been documented following prolonged administration of these chemotherapeutic agents (Farrow et al., 1997).

To date, no adjunctive pharmacological agent has been found capable of safeguarding liver function or preserving hepatic function despite continued chemotherapy. Consequently, there is an imperative for the development of a hepatoprotective agent to facilitate the optimal dosing of chemotherapy without necessitating a reduction in the prescribed doses, thereby enhancing the survival rates of pediatric ALL patients. Many herbal medicines, including silymarin, andrographolide, and glycyrrhizin, exhibit multiple protective mechanisms, such as antioxidant, free radical scavenging, antiviral, and anti-inflammatory properties, and are promising alternatives to synthetic drugs for the treatment of liver diseases, which are the leading cause of death worldwide. Recent clinical trials have demonstrated the potential of natural phytoconstituents for treating various liver diseases (Maqbool et al., 2019; Pandey et al., 2023).

Lcarnitine is a conditionally essential amino acid, and its deficiency can impair the use of fat as fuel and reduce the availability of energy in vital organs, especially the liver. The protective effects of Lcarnitine against certain liver disorders have been described in previous studies (Cave et al., 2008; Hatamkhani et al., 2014).

In light of the fact that liver diseases and the subsequent increase in liver enzymes are among the most frequent complications observed in leukemia patients who are treated with chemotherapeutic drugs (Meir et al., 2001; Shahriari et al., 2020), this study aimed to assess the impact of oral Lcarnitine supplementation on liver enzyme levels in the maintenance phase of treatment for pediatric ALL patients.

## 2 Methods and materials

### 2.1 Study subjects

This double-blind, randomized study was conducted from May 2023 to July 2024. A total of 129 ALL patients aged 5 years and older in the maintenance phase participated in the study at Ali Asghar Children’s Hospital, Tehran, Iran, with a 20% dropout rate anticipated for each group.

At the beginning of the study, a checklist was utilized to gather demographic and basic clinical data for all patients, including age, gender, body mass index (BMI), alanine aminotransferase (ALT), aspartate transferase (AST), alkaline phosphatase (ALP), gamma-glutamyl transferase (GGT), direct bilirubin, total bilirubin, prothrombin time (PT), and partial thromboplastin time (PTT).

**FIGURE 1:**
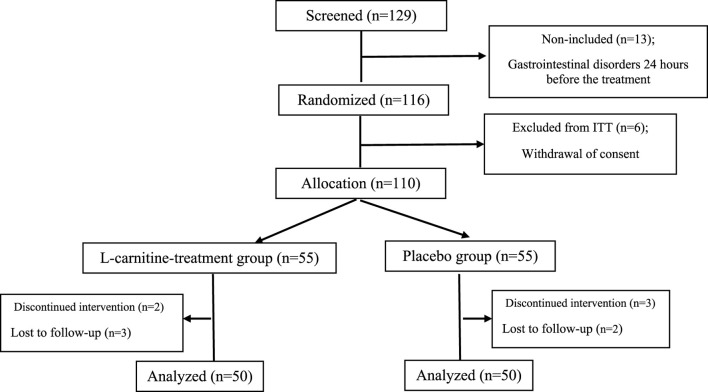
Flow chart of study procedure. ITT; intent-to-treat population. **(A)** alkaline phosphatase, **(B)** alanine aminotransferase, **(C)** aspartate transferase, **(D)** direct bilirubin, **(E)** gamma-glutamyl transferase, **(F)** prothrombin time, **(G)** total bilirubin, **(H)** partial thromboplastin time.

The sample size was determined using SPSS 25.0 software (SPSS, Inc., Chicago, IL, USA) to achieve 80% study power, a type one error of 5%, and a 95% statistical significance (P = 0.05). A biostatistician conducted randomization (allocation ratio 1:1) using a computerized random number table based on a simple randomization method inside the clinic. Consequently, the patients were randomly assigned to an Lcarnitine group and a placebo group. The study’s flowchart can be seen in Figure 1.

### 2.2 Inclusion and exclusion criteria

The inclusion criteria for this study encompassed patients aged over 5 years diagnosed with ALL who were in the maintenance phase. Individuals who indicated their willingness to participate in the research and did not have a history of any other hematologic or liver conditions, such as COVID-19, Epstein-Barr virus, hepatitis B, hepatitis C, or human immunodeficiency virus, were considered eligible. Additionally, patients with elevated liver enzymes (AST and ALT levels of 60 or higher), total bilirubin levels of 2 or more, as well as those with diabetes, a history of smoking, hypertension, cardiovascular diseases, cholecystectomy, active infections, or GGT levels of 60 or above, were excluded. Furthermore, individuals who withdrew their consent to participate in the study were also excluded. All patients were in the maintenance phase of chemotherapy and were treated with methotrexate and 6-mercaptopurine.

### 2.3 Study intervention

In the Lcarnitine group, patients received 50 mg/kg Lcarnitine syrup three times a day (every 8 h). For the control group, placebo syrup was similar to Lcarnitine in terms of dosage, shape, and color. Lcarnitine and placebo were labeled by nurses with blue and red markers, respectively, so researchers and patients were blinded to treatment allocation until the end of the study. Then, based on the randomization program, the nurse distributed colored syrups to the patients by assigning an identification code to each patient. Parents were asked to be careful when taking medicine and not to stop taking medicine for any reason without consulting a doctor. Parents were also asked to record the number of supplements taken to determine adherence to treatment. Patients were treated for a period of 2 months, and every month, assessments of the liver enzymes mentioned above were performed, and the information was recorded. Both groups were examined for liver enzymes up to 1 month after drug administration, i.e., until the end of the third month from the start of the intervention.

### 2.4 Statistical analysis

For numerical variables, data were expressed as mean ± standard deviation. Pearson’s χ2 test was used to compare categorical variables between the two groups. The mean serum AST, ALT, ALP, direct bilirubin, total bilirubin, PT, PTT, and GGT before and after intervention were compared within groups using the independent sample *t*-test. Statistical analyses were carried out using SPSS 25.0 software (Inc., Chicago, IL, USA) and a genetic analyzer (ABI PRISM 310, Applied Biosystems). P-values <0.05 were considered statistically significant.

## 3 Results

During the period of this clinical trial study, a total of 100 pediatric ALL patients were analyzed for the trial, of whom 50 patients were in the Lcarnitine treatment group and 50 patients were in the placebo group, as presented in the CONSORT diagram (Figure 1). During the study, two patients in the Lcarnitine group and three patients in the placebo group were excluded from the study due to gastrointestinal disorders 24 h before the treatment. Four patients also stopped taking the medicine due to the recommendation of their relatives, reading the brochure, and knowing the side effects of the medicine and were not willing to continue cooperation. Finally, 100 patients completed the study.

No significant difference was observed in terms of gender, age, BMI, and underlying disease severity between the two groups (P > 0.05). The mean age (±SD) in the Lcarnitine treatment group and placebo group was 7.7 ± 2.3 and 7.9 ± 2.3 years, respectively. The minimum and maximum ages in the Lcarnitine and placebo groups were 5–12 and 5–14 years, respectively (Table 1). In total, 57 patients (57.0%) were male and 43 (43.0%) were female (Table 1).

**TABLE 1:** Demographic characteristics and baseline clinical parameters of patients.

Characteristics	Lcarnitine group (N = 50)	Placebo group (N = 50)	*P*_value_
Mean age ±SD, yrs	7.7 ± 2.3	7.9 ± 2.3	0.723^b^
Gender, n (%)			
Male	25 (50.0)	32 (64.0)	0.157^a^
Female	25 (50.0)	18 (36.0)	
Mean body mass index ±SD, Kg/m2	24.4 ± 4.4	24.9 ± 4.1	0.882^b^
Mean AST ±SD, (IU/L)	141.1 ± 45.5	136.2 ± 39.7	0.570^b^
Mean ALT ±SD, (IU/L)	139.5 ± 39.3	135.1 ± 45.7	0.609^b^
Mean ALP ±SD, (IU/L)	449.2 ± 73.7	545.8 ± 128.8	**< 0.001** ^**b**^
Mean GGT ±SD, (IU/L)	79.9 ± 12.7	73.2 ± 11.1	**0.005** ^**b**^
Mean total bilirubin ±SD, mg/dL	1.58 ± 0.1	1.56 ± 0.2	0.635^b^
Mean direct bilirubin ±SD, mg/dL	0.21 ± 0.05	0.20 ± 0.05	0.552^b^
PT, seconds	12.9 ± 0.2	12.9 ± 0.2	0.996^b^
PTT, seconds	28.8 ± 1.9	28.5 ± 1.9	0.475^b^

SD; standard of deviation, n; number, ALT; alanine aminotransferase, AST; aspartate transferase, ALP; alkaline phosphatase, GGT; gamma-glutamyl transferase, PT; prothrombin time, PTT; partial thromboplastin time.

^a^Pearsonʼs χ2 test was used, ^b^Student t-test was used and Bold indicates p < 0.05.

The assessment of the consumption of Lcarnitine supplementation revealed that patients utilized over 94.1% of the prescribed supplements, thereby reflecting a significant degree of adherence to the therapeutic regimen. Overall, all patients exhibited good tolerance to Lcarnitine supplementation. Nonetheless, guardians reported the occurrence of mild gastrointestinal symptoms in a subset of patients; however, none of these symptoms were severe, and they resolved spontaneously within a few days.

Also, no significant difference was observed in terms of AST, ALT, bilirubin, PT, and PTT between the two groups (P > 0.05), although there was a significant difference in terms of ALP and GGT between the two groups at the beginning of the study (P < 0.05).

**FIGURE 2:**
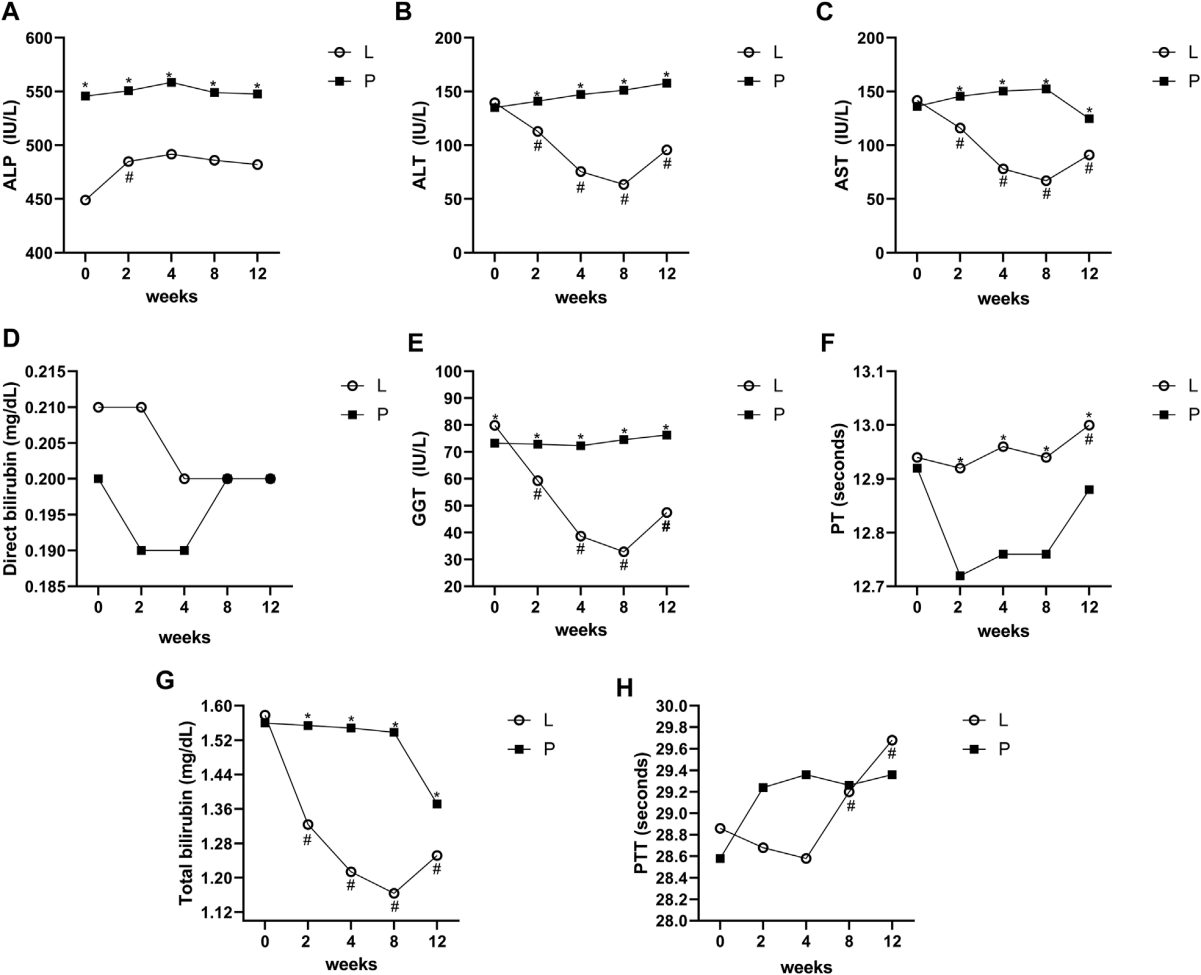
Mean values of ALT, AST, ALP, GGT, direct bilirubin, total bilirubin, PT, and PTT before and after intervention in the studied groups. ALT; alanine aminotransferase, AST; aspartate transferase, ALP: alkaline phosphatase, GGT; gamma-glutamyl transferase, PT; prothrombin time, PTT; partial thromboplastin time. *Significant difference in the comparison between the Lcarnitine group and the placebo group for each month point (point 0 vs 0 to point 5 vs 5), # significant difference in the comparison between different points of the week and the baseline point (point 1 vs 0 to point 5 vs 0) in the Lcarnitine group.

The level of the studied variables in four different periods in the Lcarnitine treatment group and placebo group is presented in Figure 2.

The mean changes of AST, ALT, total bilirubin, and GGT during the study period were significant in the group treated with Lcarnitine (P < 0.05), although they were not significant in the placebo group (P > 0.05). Also, the levels of ALP, direct bilirubin, PT, and PTT were not significantly different between the two groups. Changes between the two groups for all variables, both overall and at each time point, are shown in Figure 2.

**FIGURE 3:**
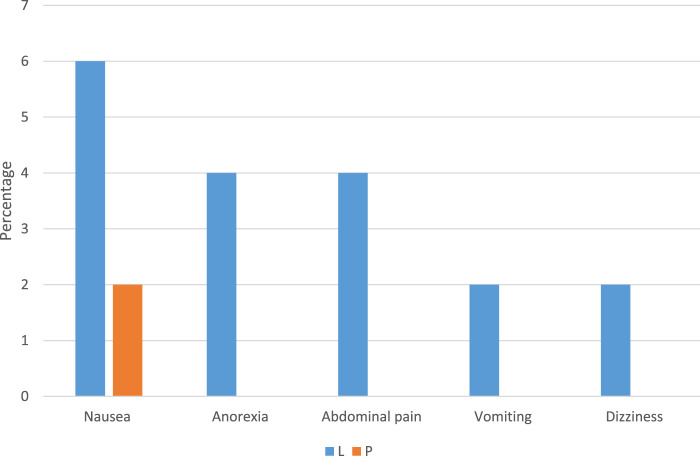
Comparison of medicine side effects among different groups of Lcarnitine recipients and placebo. Data are reported as percentage, L: Lcarnitine group, P: placebo group.

The results of the assessment of medication side effects in two groups are shown in Figure 3. The incidence of side effects in the Lcarnitine group was significantly higher than in the placebo group (18% vs 4%, P = 0.025). Side effects observed in the Lcarnitine group included nausea (3 patients), anorexia (2 patients), abdominal pain (2 patients), vomiting (1 patient), and dizziness (1 patient). Only one side effect was observed in the placebo group, which included nausea (2 patients).

## 4 Discussion

Leukemia is one of the most common malignancies encountered within the pediatric population, affecting an estimated 40 children per million among individuals aged below 29 years (Howlader et al., 2013). Numerous studies have revealed a notable incidence of hepatotoxicity in pediatric ALL patients (Denton et al., 2018; Christ et al., 2018). Moderate hepatic dysfunction is often observed in both pediatric and adult populations diagnosed with ALL (Baillargeon et al., 2005). Segal et al. performed an extensive analysis involving 147 children diagnosed with ALL from 2001 to 2006 (Segal et al., 2010). In agreement with our research findings, their investigation indicated that elevated liver transaminases and hyperbilirubinemia were common clinical manifestations in pediatric ALL patients. This finding highlights the imperative need for the identification of effective pharmacological interventions to alleviate hepatotoxicity in pediatric ALL patients.

Lcarnitineis an essential amino acid that plays an important role in cellular energy metabolism, and its deficiency is associated with liver failure (Hatamkhani et al., 2014; Inazu and Matsumiya, 2008).

Contemporary randomized controlled trials (RCTs) have evaluated the impact of Lcarnitine supplementation on hepatic parameters by examining its associated enzymes. Nevertheless, these outcomes are contradictory, and no research to date has established a conclusive determination in this area. Among the RCTs incorporated within the present systematic review and meta-analysis, the majority of investigations demonstrated a statistically significant decrease in hepatic enzyme levels after Lcarnitine supplementation, although a limited subset of studies reported no notable effect. These divergent findings may be attributable to variations in the methodological rigor of the included RCTs or the differing health status of the participants.

The aim of this clinical research was to investigate the effect of Lcarnitine syrup on liver enzyme levels in pediatric ALL patients who were undergoing chemotherapy in the maintenance phase. Our findings indicate that Lcarnitine can be safely administered to children receiving maintenance therapy for ALL. The provision of a 2-month regimen of Lcarnitine was correlated with a statistically significant reduction in ALT, AST, GGT, and total bilirubin levels. Notably, no reductions in chemotherapy dosage, atypical toxicities, or treatment interruptions were observed during Lcarnitine administration. Consistent with our results, Aldoss et al. showed that Lcarnitine can also be used to accelerate the normalization of enzyme levels and prevent the postponement of subsequent cycles of chemotherapy (Aldoss and Douer, 2020).

In addition, in a study conducted on pediatric ALL patients, the significant effect of using Lcarnitine on the reduction of liver enzyme levels in weeks 10 and 12 after the intervention was shown (Golpayegani et al., 2021). In another study, Hazzan et al. revealed the hepatoprotective effects of Lcarnitine in patients with non-alcoholic fatty liver disease, showing that Lcarnitine combined with magnesium may be a potential therapy for reducing liver enzyme levels in non-alcoholic fatty liver disease patients (Hazzan et al., 2022). Recent clinical trials have shown that carnitine supplementation can effectively address liver cirrhosis complications, including hepatic encephalopathy, sarcopenia, and muscle cramps, potentially improving patients' quality of life (Hanai et al., 2020).

Several herbal medicines, including Silybum marianum and Glycyrrhiza glabra, exert hepatoprotective effects through immunomodulatory and antioxidant activity (Ali et al., 2018). Turmeric and vitamin C, both individually and in combination, attenuated lead acetate-induced liver injury in rats by reducing oxidative stress, regulating Bax and Bcl-2 protein expressions, and decreasing DNA damage (Alhusaini et al., 2019).

The beneficial effects of Lcarnitine supplementation on oxidative stress have been previously reported. Lcarnitine has very important physiological roles, including the transfer of long-chain fatty acids from the cytoplasm to the mitochondria in liver cells, and as a result, it increases the beta-oxidation of these fatty acids. Therefore, Lcarnitine supplementation may affect liver function (Mortazavi et al., 2011; Adeva‐Andany et al., 2017).

In our study, the percentage of side effects was significantly higher in the Lcarnitine group than in the placebo group, whereas Schulte et al. showed that the use of Lcarnitine in patients with ALL was easily tolerated by the patients, and it has no side effects or adverse interactions with chemotherapy (Schulte et al., 2021). Similar studies have reported gastrointestinal symptoms and mild issues like dizziness as common side the effects of Lcarnitine supplementation. However, these side effects are generally transient and tend to subside over time (Goin-Kochel et al., 2019). One of the reasons for the difference in the results can be due to the age of the studied patients. The higher incidence of side effects in the Lcarnitine group may be related to the pharmacological effects of the supplement and the characteristics of the target population. These findings are consistent with those of previous studies and indicate the relative safety of the supplement with manageable side effects (Vasiljevski et al., 2021).

The study’s limitations include its short duration, small sample size, and variability in liver enzyme responses, which may have been influenced by individual factors. It is recommended that longitudinal studies with larger and more diverse populations be conducted to better understand the long-term effects and mechanisms underlying this variability.

## 5 Conclusion

In conclusion, the results of this study suggested that 60-day Lcarnitine treatment can improve liver enzyme levels and thus prevent the extent of liver damage during the treatment of ALL. The side effects of Lcarnitine are tolerable in pediatric ALL patients. Therefore, Lcarnitine can be used as an adjuvant therapy for these patients. Based on the results of our study, Lcarnitine supplementation may have a beneficial effect on liver enzyme levels in pediatric ALL patients during the maintenance phase of treatment.

## Data Availability

The raw data supporting the conclusions of this article will be made available by the authors, without undue reservation.
